# Comparative Label-Based Proteomics of Venoms from *Echis ocellatus*, *Naja nigricollis*, and *Bitis arietans*

**DOI:** 10.3390/proteomes13030031

**Published:** 2025-07-02

**Authors:** Abdulbaki Alfa-Ibrahim Adio, Samuel Odo Uko, Jiddah Muhammad Lawal, Ibrahim Malami, Nafiu Lawal, Amina Jega Yusuf Jega, Bilyaminu Abubakar, Muhammad Bashir Bello, Kasimu Ghandi Ibrahim, Murtala Bello Abubakar, Abdussamad Muhammad Abdussamad, Mujtaba Sulaiman Abubakar, Mustapha Umar Imam

**Affiliations:** 1 Centre for Advanced Medical Research and Training, Usmanu Danfodiyo University, Sokoto P.M.B. 2346, Nigeria; abdulbakiibrahim27@gmail.com (A.A.-I.A.); ukosam10@gmail.com (S.O.U.); ibrahim.malami@udusok.edu.ng (I.M.); nafiu.lawal@udusok.edu.ng (N.L.); amina.yusuf@udusok.edu.ng (A.J.Y.J.); abubakar.bilyaminu@udusok.edu.ng (B.A.); bbtambuwal@gmail.com (M.B.B.); 2 Department of Biochemistry and Molecular Biology, Faculty of Chemical and Life Sciences, Usmanu Danfodiyo University, Sokoto P.M.B. 2346, Nigeria; 3 Department of Pharmacognosy and Ethnopharmacy, Faculty of Pharmaceutical Sciences, Usmanu Danfodiyo University, Sokoto P.M.B. 2346, Nigeria; 4 Department of Veterinary Microbiology, Faculty of Veterinary Medicine, Usmanu Danfodiyo University, Sokoto P.M.B. 2346, Nigeria; 5 Department of Pharmaceutical and Medicinal Chemistry, Faculty of Pharmaceutical Sciences, Usmanu Danfodiyo University, Sokoto P.M.B. 2346, Nigeria; 6 Department of Pharmacology and Toxicology, Faculty of Pharmaceutical Sciences, Usmanu Danfodiyo University, Sokoto P.M.B. 2346, Nigeria; 7 Infectious Disease Research Development, King Abdullah International Medical Research Center, Riyadh 11481, Saudi Arabia; 8 Department of Basic Medical and Dental Sciences, Faculty of Dentistry, Zarqa University, P.O. Box 2000, Zarqa 13110, Jordan; kibrahim@zu.edu.jo; 9 Department of Physiology, College of Medicine and Health Sciences, Sultan Qaboos University, Muscat 123, Oman; m.abubakar@squ.edu.om; 10 Department of Veterinary Physiology and Biochemistry, Faculty of Veterinary Medicine, Bayero University, Kano P.M.B. 3011, Nigeria; amabdussamad.vpb@buk.edu.ng; 11 Department of Pharmacognosy & Drug Development, Faculty of Pharmaceutical Sciences, Ahmadu Bello University, Zaria P.M.B. 1045, Nigeria; msabubakar@abu.edu.ng; 12 Venom, Antivenom & Natural Toxins Research Centre, Ahmadu Bello University, Zaria P.M.B. 1045, Nigeria; 13 Department of Medical Biochemistry, Faculty of Basic Medical Sciences, College of Health Sciences, Usmanu Danfodiyo University, Sokoto P.M.B. 2346, Nigeria

**Keywords:** snake venom, proteomics, iTRAQ, mass spectrometry, envenomation, antivenom development

## Abstract

**Background:** Snake envenomation is a major public health issue in Nigeria, primarily due to bites from *Echis ocellatus*, *Naja nigricollis*, and Bitis arietans. Understanding their venom composition is essential for effective antivenom development. This study characterizes and compares the venom proteomes of these snakes using iTRAQ-based proteomics, focusing on key toxin families and their relative abundances. **Methods:** Venom samples were ethically collected from adult snakes, pooled by species, lyophilized, and stored for proteomic analysis. Proteins were extracted, digested with trypsin, and labeled with iTRAQ. Peptides were analyzed via mass spectrometry, and data were processed using Mascot and IQuant for protein identification and quantification. **Results:** *E. ocellatus* and *B. arietans* venoms had similar profiles, rich in C-type lectins, serine proteases, and phospholipase A_2_s. These comprised 17%, 11%, and 5% in *E. ocellatus* and 47%, 10%, and 7% in *B. arietans*, with metalloproteinases dominating both (53% and 47%). In *N. nigricollis*, three-finger toxins (9%) were most abundant, followed by metalloproteinases (3%). All species shared four core protein families, with *N. nigricollis* also containing four uncharacterized proteins. **Conclusions:** This study highlights venom compositional differences, advancing snake venom biology and informing targeted antivenom development.

## 1. Introduction

Snake envenomation is a critical public health issue, particularly in tropical and subtropical regions where snakebite-related morbidity and mortality are high. According to the World Health Organization (WHO), an estimated 5.4 million people are bitten by snakes annually, resulting in 1.8 to 2.7 million cases of envenomation and around 81,000 to 138,000 deaths worldwide [1]. Nigeria, in particular, is heavily affected, with *Echis ocellatus*, *Naja nigricollis*, and *Bitis arietans* being the most medically significant species responsible for numerous envenomation cases [2,3]. *E. ocellatus* is the leading cause of snakebite mortality in Bauchi State and the Benue Valley, with high incidence rates (497 per 100,000 annually) reported in these regions [4]. Similarly, *N. nigricollis* and *B. arietans* are distributed among rural dwellers in the Savanna regions of the country [4]. Current treatments rely on polyvalent antivenoms, which often lack specificity to critical toxins due to incomplete understanding of venom compositions [5]. For instance, *E. ocellatus* venom causes life-threatening coagulopathy, but antivenom may not fully neutralize region-specific procoagulant enzymes [6]. Proteomics analysis may, however, be crucially needed to understand and identify venom proteins to design species- and region-specific antivenoms, ensuring precise toxin neutralization.

Proteomics, the large-scale study of proteins, is essential for characterizing venom composition. Traditional methods, such as biochemical assays and immunological techniques, often fail to capture the complexity of venom proteomes, which contain hundreds of proteins with diverse functions. Modern mass spectrometry (MS)-based approaches, however, allow for more comprehensive analyses of venom proteins, enabling the identification and quantification of numerous toxins simultaneously [7,8]. These proteomic studies are crucial in understanding venom diversity, the molecular mechanisms behind envenomation, and the evolution of venomous species.

Among MS-based techniques, Isobaric Tags for Relative and Absolute Quantitation (iTRAQ) has proven to be an invaluable tool for venom proteomics [8]. This technique (iTRAQ) has gained widespread popularity as a technique used to estimate protein quantitation from MS data. Like other established mass spectrometry (MS)-based quantitative techniques such as isotope coded affinity tag (ICAT), iTRAQ technology involves stable-isotope labeling, either through chemical modification of proteins or peptides for the detection of differential regulation [9,10]. While Tandem Mass Tag (TMT)—another common MS-based in vitro labeling technique—can be used to compare samples by labeling them with alternative differential mass tags, thus allowing detection based on specific change in mass, iTRAQ stands out in many ways as an effective and power labeling technique for proteomics analysis and quantification of venom proteins. For instance, iTRAQ has a longer track record in venom/toxin research than TMT. To date, nearly all published isobaric quantitation in snake venomics has used iTRAQ. Notably, Zelanis et al. (2011) used iTRAQ to quantify differential toxin abundance in *B. jararaca* venoms [11], and Gao et al. (2013) used it in *G. brevicaudus* [12].

These pioneering studies identified hundreds of toxin peptides and demonstrated that iTRAQ can robustly profile entire venom proteomes under varied conditions. By contrast, TMT-based profiling of snake venoms remains rare, so iTRAQ benefits from validated protocols and accumulated experience in the toxinology community. In all these cases, iTRAQ quantification revealed fine-scale differences in toxin families (e.g., metalloproteases and phospholipases), showing that the method provides thorough coverage of venom complexity [11,12]. Moreover, iTRAQ data analysis workflows (normalization, ratio calculation) are well integrated into proteomic software. In summary, iTRAQ offers a proven, well-supported platform for venom proteomics, with extensive literature precedent in snake toxin research.

iTRAQ also enables the simultaneous comparison of protein abundance across multiple samples by tagging peptides with isobaric labels [13]. During MS analysis, these tags produce unique reporter ions upon fragmentation, which are then used to quantify the peptides from different samples [14]. In snake venom research, iTRAQ offers a powerful advantage by allowing the relative quantification of proteins from multiple species or geographical populations, facilitating the identification of key toxins and differences in venom composition [13]. In contrast, traditional techniques like 2D-GE suffer limitations such as labor-intensiveness and reproducibility issues, particularly with complex samples like venom proteins [13]. While label-free quantification is another option, it lacks the precision and multiplexing capabilities offered by iTRAQ, making it less suitable for large-scale venom studies. For these reasons, iTRAQ is the preferred technology for this research, allowing for a comprehensive, high-throughput, and quantitative analysis of venom proteomes.

In this study, we employed iTRAQ-based proteomics to characterize the venoms of *Echis ocellatus*, *Naja nigricollis*, and *Bitis arietans*—the three major snakes responsible for envenomation in Nigeria. By utilizing iTRAQ technology, we aim to gain deeper insights into the proteomic profile of these snake species and compare their venom proteomes. It is also our goal to identify and compare the major toxin families and proteins responsible for their venom’s pathological effects. This analysis will provide comprehensive data that will inform the development of more effective antivenoms for treating envenomation caused by these snakes.

## 2. Materials and Methods

### 2.1. Snake Venom Samples

Snake venom samples were collected from *N. nigricollis*, *E. ocellatus*, and *B. arietans* as per ethical guidelines. The handling protocols were approved by the Usmanu Danfodiyo University Committee on Animal Use and Care and complied with the ARRIVE guidelines [15]. Adult snakes of each species were housed in the Herpetorium at Ahmadu Bello University, Zaria. Venom samples were lyophilized immediately after collection and stored at 4 °C for approximately four weeks prior to use.

### 2.2. Chemicals and Reagents

The iTRAQ 4plex reagent kit was used for isobaric labeling with standard marker proteins and analytical-grade trypsin (Bioseutica^®^ group, Zeewolde, The Netherlands). All other chemicals and reagents, including those used in protein extraction and mass spectrometry, were of analytical grade to maintain the integrity of the experimental results.

### 2.3. Venom Protein Extraction and Solubilization

Two milligrams of sample (1xCocktail) was added to EDTA (without SDS) and placed on ice for 5 min, followed by the addition of DTT to a final concentration of 10 mM. The sample was sonicated and centrifuged at 25,000× *g* at 4 °C for 15 min, with the supernatant collected as the protein solution. The supernatant was incubated with 10 mM DTT at 56 °C for 1 h to reduce disulfide bonds, followed by alkylation with 55 mM iodoacetamide (IAM) in a dark room for 45 min to prevent reformation of disulfide bonds. The mixture was then centrifuged again at 25,000× *g* at 4 °C for 15 min to collect the final protein solution.

### 2.4. Quality Control of Protein Extraction

The protein concentration was quantified using the Bradford assay [16]. Standard proteins (0.2 μg/μL BSA) were serially diluted and added to a 96-well plate (0 to 18 μL), followed by the addition of Coomassie Brilliant Blue G-250 solution. The optical density was measured at 595 nm using a microplate reader, and a standard curve was constructed based on the protein concentration and absorbance. Samples were diluted and measured similarly, and their concentrations were determined using the standard curve.

### 2.5. One-Dimensional SDS-PAGE

Each 10 μg of venom protein was mixed with loading buffer and heated at 95 °C for 5 min to denature the proteins. The samples were then centrifuged at 25,000× *g* for 5 min, and the supernatant was loaded into a 12% SDS-polyacrylamide gel for electrophoresis. The gel was run at 80 V for 30 min, followed by 120 V for 120 min. After electrophoresis, the gel was stained with Coomassie Brilliant Blue and de-stained in a solution containing 10% glacial acetic acid and 1% glycerol. The stained gel was scanned for further analysis.

### 2.6. In-Solution Protein Digestion

For protein digestion, 100 μg of the extracted protein was transferred to a 1.5 mL centrifuge tube. The protein solution was diluted with 50 mM triethylammonium bicarbonate (TEAB) buffer to provide an optimal environment for enzymatic digestion. Trypsin was added at an enzyme-to-protein ratio of 1:20 (*w*/*w*) and the mixture was vortexed gently, centrifuged briefly at low speed, and incubated at 37 °C for 4 h to achieve complete proteolysis. After digestion, the resulting peptides were desalted using C18 spin columns and subsequently freeze-dried for downstream iTRAQ labeling.

### 2.7. Isobaric Peptide Labeling

For iTRAQ labeling, the dried peptides were reconstituted in 0.5 M TEAB. The iTRAQ reagent was allowed to reach room temperature before the addition of 50 μL isopropanol. The peptides were labeled with isobaric tags, vortexed, and left at room temperature for 2 h to ensure complete labeling. This labeling allows for the multiplexing of samples and their comparative quantification in the mass spectrometry analysis [17]. This study was conducted using a single iTRAQ experiment without technical or biological replicates. Nonetheless, rigorous quality control procedures and validated quantification methods were employed to ensure data reliability. Moreover, Karp et al. [18] and Mertins et al. [19] have both reported the reproducibility of iTRAQ experiments, highlighting consistency and low technical variability between samples.

### 2.8. Peptide Fractionation

Peptide fractionation was carried out using a Shimadzu LC-20AB system (Shimadzu Corporation, Kyoto, Japan). The separation column was a 5 µm 4.6 × 250 mm Gemini C18 column for liquid phase separation of the sample. The dried peptide samples were reconstituted with mobile phase A (5% ACN pH 9.8) and injected, eluting at a flow rate of 1 mL/min by the following gradients: 5% mobile phase B (95% ACN, pH 9.8) for 10 min, 5% to 35% mobile phase B for 40 min, 35% to 95% mobile phase B for 1 min, mobile phase B for 3 min, and 5% mobile phase B for 10 min. The elution peak was monitored at a wavelength of 214 nm and one component was collected per minute, and the samples were combined according to the chromatographic elution peak map to obtain 20 fractions, which were then freeze-dried.

### 2.9. Liquid Chromatography

The peptide samples were reconstituted with mobile phase A (2% ACN, 0.1% FA), centrifuged at 20,000× *g* for 10 min, and the supernatant was taken for injection. Separation was performed by Thermo UltiMate 3000 UHPLC (Thermo Scientific, Waltham, MA, USA). The sample was first enriched in the trap column and desalted, and then entered a self-packed C18 column (75 μm internal diameter, 3 μm column size, 25 cm column length) and separated at a flow rate of 300 nL/min by the following effective gradients: 0~5 min, 5% mobile phase B (98% ACN, 0.1% FA); 5~45 min, mobile phase B linearly increased from 5% to 25%; 45~50 min, mobile phase B increased from 25% to 35%; 50~52 min, mobile phase B rose from 35% to 80%; 52~54 min, 80% mobile phase B; 54~60 min, 5% mobile phase B. The nanoliter liquid phase separation end was directly connected to the mass spectrometer.

### 2.10. Mass Spectrometry (MS/MS) Analysis

The peptides separated by liquid phase chromatography were ionized by a nanoESI source and then passed to a tandem mass spectrometer Q-Exactive HF X (Thermo Fisher Scientific, San Jose, CA, USA) for DDA (Data Dependent Acquisition) mode detection. The main parameters were set as follows: ion source voltage was set to 1.9 kV, MS1 scanning range was 350~1500 *m*/*z*; resolution was set to 60,000; MS2 starting *m*/*z* was fixed at 100; resolution was 15,000. The ion screening conditions for MS2 fragmentation were as follows: charge 2+ to 6+, and the top 30 parent ions with the peak intensity exceeding 10,000. The isolation width was set to 1.6 Da, and the maximum injection time was set to 100 ms. The MS2 ion fragmentation mode was performed using higher-energy collisional dissociation (HCD), and the fragment ions were detected in Orbitrap. The normalized collision energy (NCE) for HCD was set to 35. The dynamic exclusion time was set to 30 s. The AGC was set to MS1 3E6 and MS2 1E5.

### 2.11. Database Search

Following LC-MS/MS analysis, tandem mass spectra were searched using Mascot MS/MS ion search (Matrix Science, London, UK) against the Uniprot-Serpentes database (available at https://www.uniprot.org) containing 31,896 sequences (up-dated in December 2023). Search parameters included: enzyme specificity set to trypsin with one allowed missed cleavage; fixed modifications as carbamidomethylation (C), iTRAQ8plex at N-terminus and lysine (K); variable modifications including oxidation (M), deamidation (NQ), and iTRAQ8plex (Y); peptide mass tolerance set to 10 ppm; fragment mass tolerance set to 0.02 Da; monoisotopic mass values were used. Only proteins with at least one unique peptide and a Mascot significance threshold of *p* < 0.05 were considered for identification and iTRAQ-based quantification.

### 2.12. Protein Quantification and Data Analysis

**Figure 1:**
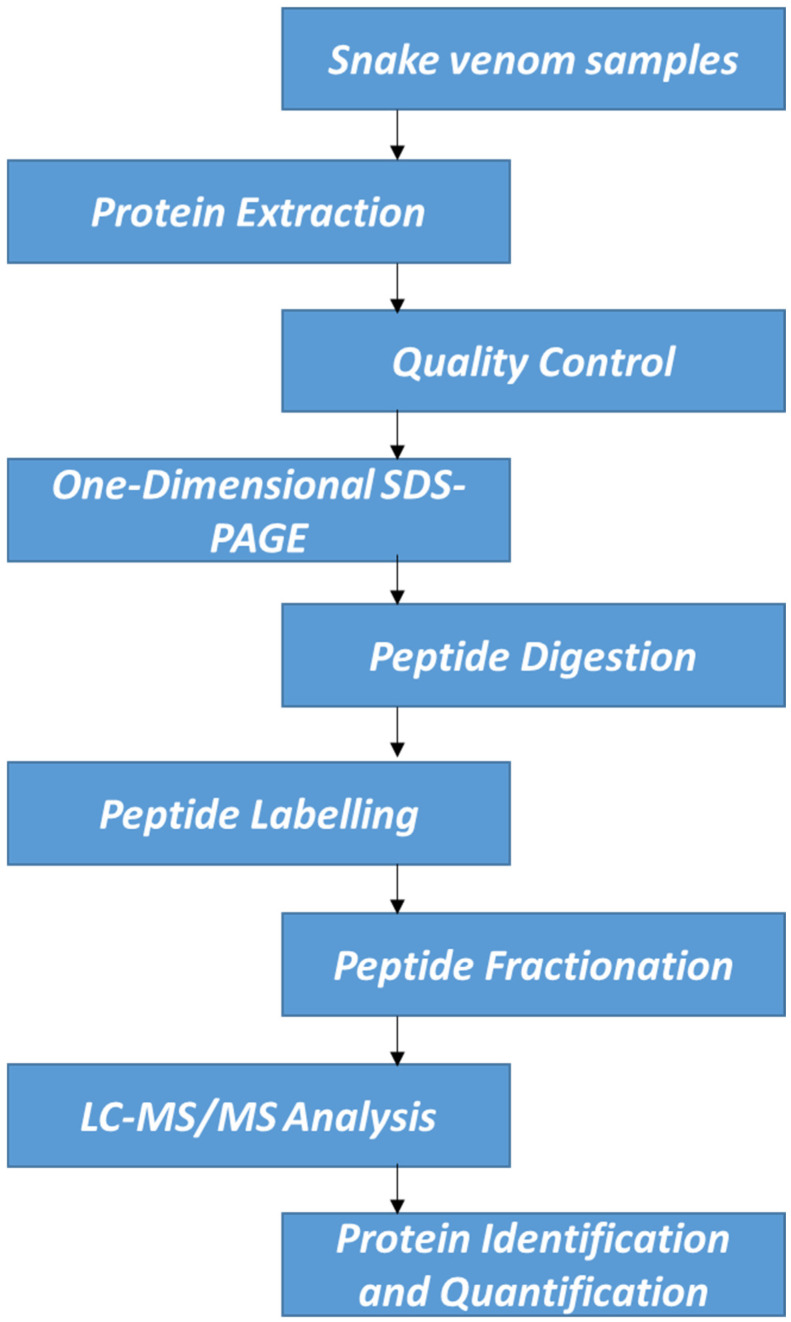
Schematic experimental workflow.

For protein quantification, IQuant version 2.0.1, an automated software tool (available at http://sourceforge.net/projects/iquant/, 24 June 2025), was employed, integrating Mascot Percolator, a machine learning method for rescoring database search results to enhance the accuracy and reliability of significance measures. At the peptide level, peptide-spectrum matches (PSMs) were pre-filtered at a false discovery rate (FDR) of 1% to ensure the confidence of peptide identification. Using the “parsimony principle,” the identified peptide sequences were then assembled into a set of confident proteins. Protein-level FDR was controlled at ≤1% (Protein FDR ≤ 0.01) using the “Picked Protein FDR” strategy after protein inference. To normalize the protein quantification data, variance stabilization normalization (VSN) was applied. A permutation test was conducted to evaluate the statistical significance of protein quantitative ratios. The final results of protein identification and quantification were exported as tab-delimited text files for subsequent analysis and interpretation. Interventionary studies involving animals or humans, and other studies that require ethical approval, must list the authority that provided approval and the corresponding ethical approval code. Although only a single iTRAQ experiment was performed, we ensured high reliability of quantification through strict peptide/protein FDR thresholds, use of Mascot Percolator, and VSN. Figure 1 demonstrates the workflow of this experimental study.

## 3. Results

### 3.1. One-Dimensional SDS-PAGE of the Crude Snake Venoms

**Figure 2:**
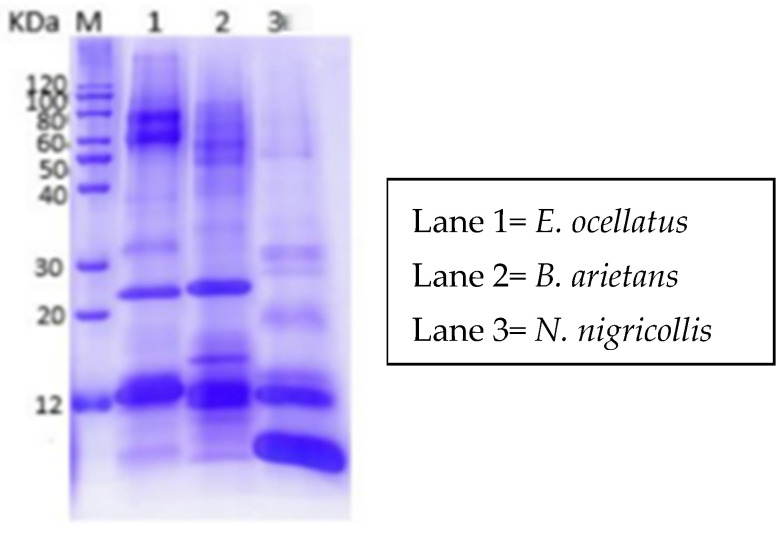
One-dimensional SDS-PAGE profile of *N. nigricollis*, *E. ocellatus* and *B. arietans*.

The results of the one-dimensional SDS-PAGE analysis of the crude venoms from *E. ocellatus*, *B. arietans*, and *N. nigricollis* displayed distinct protein band patterns, reflecting differences in their protein compositions (Figure 2). The venoms of *E. ocellatus* and *B. arietans* revealed similar protein banding patterns, with prominent bands observed at molecular weight ranges of approximately 12 kDa, 25 kDa, and 100 kDa. This similarity in banding can be attributed to their phylogenetic relationship, as both species belong to the *Viperidae* family. Viperid venoms are known to share several common protein families, such as snake venom metalloproteinases (SVMPs), snake venom serine proteases (SVSPs), and phospholipases A_2_ (PLA_2_s), which contribute to their hemotoxic and proteolytic effects [20].

In contrast, the venom of *Naja nigricollis* showed a different banding profile, with proteins revealing between <12 kDa and 60 kDa. This venom’s distinct pattern is indicative of the unique composition of elapid venoms, which are rich in neurotoxins and cytotoxins. The absence of high-molecular-weight bands, such as those seen in *E. ocellatus* and *B. arietans*, is characteristic of elapid venoms, which typically contain smaller proteins that target the nervous system rather than causing extensive tissue damage. The differences in protein profiles between *N. nigricollis* and the viperids (*E. ocellatus* and *B. arietans*) demonstrate the heterogeneous nature of snake venoms. While viperid venoms are generally more hemotoxic, targeting blood clotting mechanisms and causing tissue necrosis, elapid venoms are primarily neurotoxic, affecting the nervous system.

### 3.2. Snake Venom Proteomic Characterization Using LC-MS/MS

**Figure 3:**
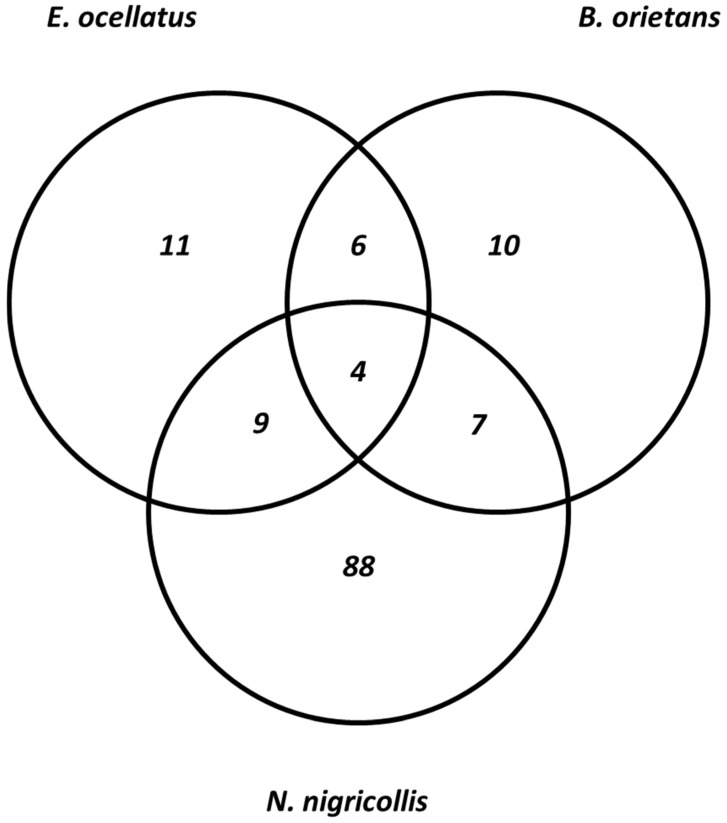
Comparison of the identified protein families across the three snake species.

The result of isobaric peptide tagging and LC-MS/MS analysis revealed a total of 240 distinct venom proteins were identified across the three species. Approximately, 139, 71, and 30 proteins were identified in the crude venom extracts of *Naja nigricollis*, *Echis ocellatus*, and *Bitis arietans*, respectively (Supplementary Table S1). The identified proteins ranged in molecular mass from 3 to 389 kDa. These proteins belong to 10, 11, and 88 toxin families in the venoms of *Bitis arietans*, *Echis ocellatus*, and *Naja nigricollis*, respectively. Notably, more common proteins were shared between *Echis ocellatus* and *Bitis arietans*, a similarity attributed to their classification within the same genus. These vipers shared five protein families: C-type lectins, SVSPs, SVMPs, PLA_2_s, and disintegrins. It is well-established that venom proteomes of snakes within the same genus often display similarities despite differences in taxonomy or geographic location [21]. In contrast, *E. ocellatus* and *N. nigricollis* shared nine proteins, while *B. arietans* and *N. nigricollis* shared seven proteins (Figure 3). These findings highlight the biochemical diversity of the venoms: elapid venoms (*N. nigricollis*) are more neurotoxic, whereas viperid venoms (*E. ocellatus* and *B. arietans*) predominantly rely on hemotoxic enzymes [22]. Table 1 presents the list of identified protein families across the three species.

### 3.3. Relative Distribution of the Protein Families

The relative distribution of these protein families is summarized in Supplementary Table S1. Metalloproteinases (SVMPs) were the most abundant protein family in *E. ocellatus* venom, having an intensity of 508.6 (53%), followed by C-type lectins with an intensity of 162.8 (17%) and serine proteinases (SVSPs) with 104.1 (11%). PLA_2_ accounted for 5% of the total protein with an intensity of 50.2. Similarly, in the venom of *B. arietans*, C-type lectins showed the highest relative abundance of 47% (193.1 intensity), followed by serine proteinases with high protein level of 40.4 (10%) and PLA_2_ with intensity of 30.8 (7%). Metalloproteinases constituted a smaller proportion, alongside disintegrins and cystatins, each representing 6% of the venom proteome. In contrast, the proteomic profile of *N. nigricollis* venom revealed that three-finger toxins (3FTxs) were the most abundant toxin family with a relative abundance of 160.8 (9%), followed by metalloproteinases with an intensity of 54.1 (3%). While this study identified a complex array of protein components in *N. nigricollis* venom, many proteins had not been previously reported in the Nigerian *N. nigricollis*. Most of these auxiliary proteins showed minimal or no toxicity. These secondary proteins were present alongside major toxins such as 3FTxs, PLA_2_s, SVSPs, SVMPs, and C-type lectins.

The large presence of auxiliary proteins in *N. nigricollis* venom likely masked the relative abundance of major toxins, resulting in the low observed percentages of 3FTxs, SVMPs, and PLA_2_s (9%, 3%, and 3%, respectively). The absence of 3FTxs in viper venoms underscores their divergent venom strategies, with viperids focusing on hemotoxicity and elapids on neurotoxicity [23].

### 3.4. Relative Distribution of Protein Families Based on Protein Sequence Coverage

**Figure 4:**
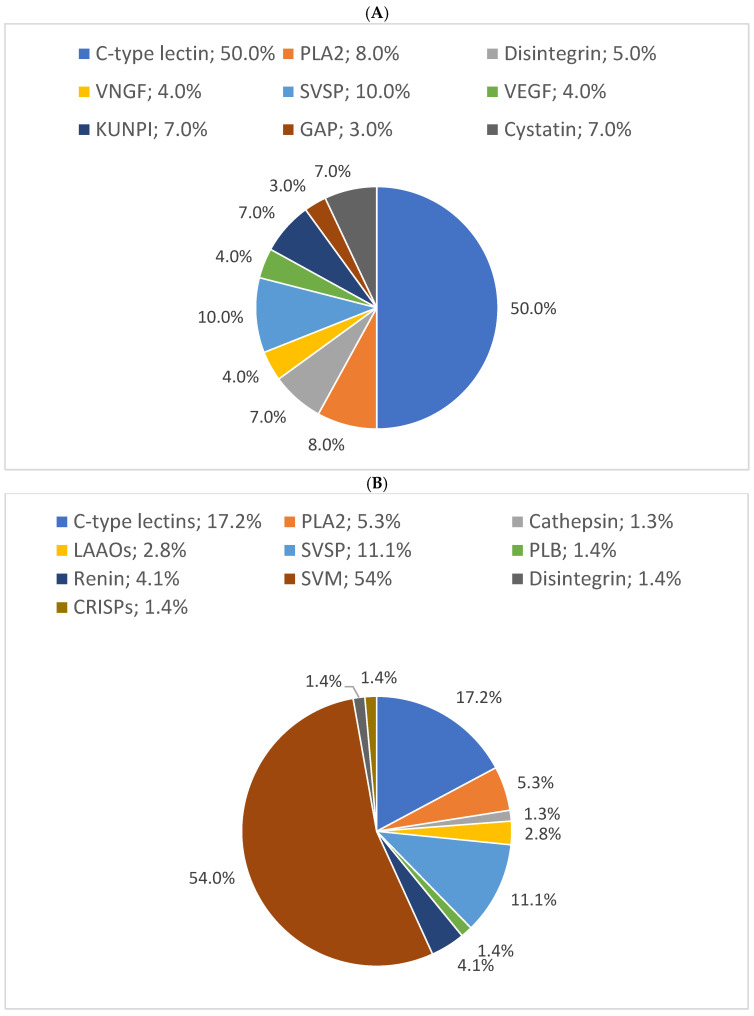

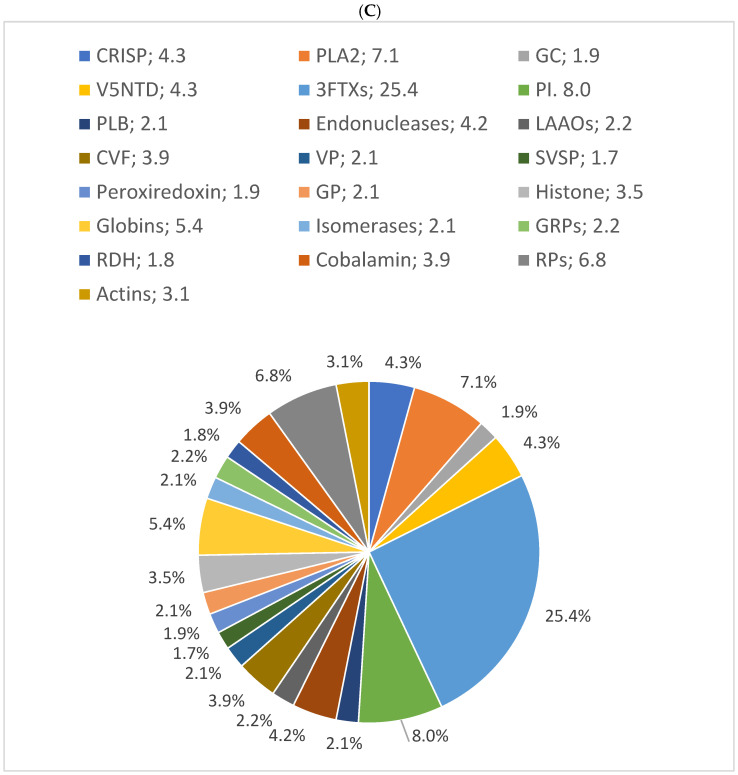
Relative distribution of (**A**) *B. arietans,* (**B**) *E. ocellatus* and (**C**) *N. nigricollis* venom protein families based on protein sequence coverage. GAP: glutamyl aminopeptidase; PLA_2_s: phospholipases A2; VNGF: venom nerve growth factor; SVSPs: snake venom serine proteases; VEGF: venom endothelial growth factor; KUNPI: Kunitz-type protease inhibitor; GAP: glutamyl aminopeptidase; LAAO: L-amino acid oxidase; SVMs: snake venom metalloproteinases; CRISP: cysteine-rich secretory protein; PIs: protease inhibitors; VP: venom phosphodiesterase; RPs: ribosomal proteins; PLB: phospholipase B; GC: glutaminyl cyclase; GRPs: glucose-regulated proteins; V5NTD: venom 5′-nucleotidases; GP: glutathione peroxidase; RDH: retinol dehydrogenase; 3FTXs: three-finger toxins; CVF: cobra venom factor.

In this study, the results showed that 54% of proteins have 10% sequence coverage. Thus, 10% was used as a threshold to classify as “high sequence coverage”. The results of the distribution of protein families with high protein sequence coverage (>10%) showed C-type lectins, SVSPs, and PLA_2_s were the most abundant protein families in *B. arietans* venom, with relative abundances of 50%, 10%, and 8%, respectively. The proteomic results for the three snakes are presented in pie charts (Figure 4).

### 3.5. Protein Mass Distribution, Unique Peptide Number and Protein Coverage Distribution

**Figure 5:**
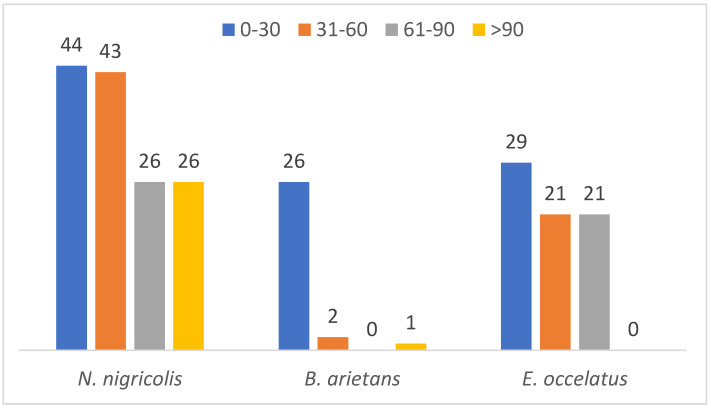
Mass distribution of identified proteins across the three snake species.

A parallel comparison of the protein mass distribution across the three species revealed that proteins with molecular masses between 0 and 30 kDa show high abundance across all the species (44, 26 and 29 in *N. nigricollis*, *B. arietans* and *E. ocellatus*, respectively). This finding suggests that the majority of the identified in this study have low molecular mass accounting for 22.5% of the detected proteins. In contrast, only 7.5% of the proteins have a mass exceeding 100 kDa (Figure 5). This distribution suggests a predominance of low- to mid-molecular-weight proteins in the venoms of the three species, which aligns with the functional versatility required for venom efficacy.

Further analysis of protein families showed significant differences in mass distribution among the species. Complement proteins, with an average mass of 353.9 kDa, are the largest protein family detected in the venom glands of *Naja nigricollis*. In *B. arietans*, C-type lectins were the largest massive family at 240.4 kDa, while in *E. ocellatus*, SVMPs dominated with an exceptionally high average molecular mass of 2135.1 kDa.

**Figure 6:**
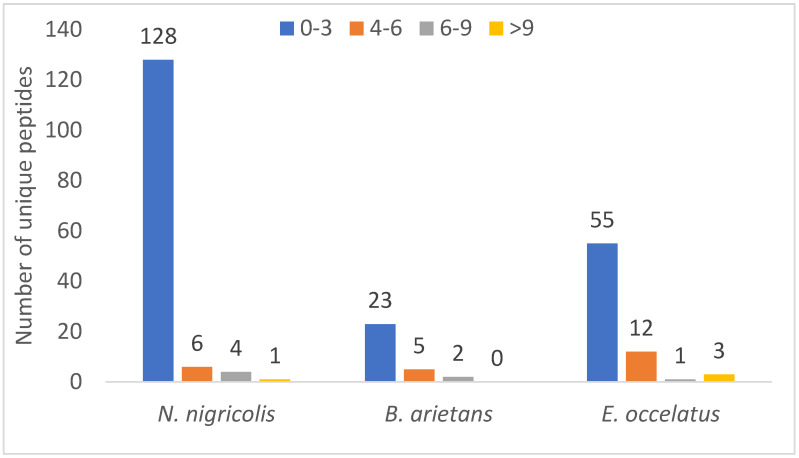
Distribution of unique peptide number across the three snake species.

Similarly, a relative comparison of unique peptide numbers, an important parameter in protein identification strategies to increase confidence, revealed notable patterns. Out of 240 identified proteins, approximately 151 (62%) were detected with just one unique peptide, and only 1% of proteins had peptides >10%. Figure 6 shows that 128, 23 and 55 proteins have 0–3 unique peptides in *N. nigricollis*, *B. arietans* and *E. ocellatus,* respectively, when compared with other ranges of unique protein numbers. It further revealed that three proteins in the venom of *E. ocellatus* were detected with more than 9 unique peptides, while only one protein was found to have 13 unique peptides as none was found with more than 9 unique peptides in the venom of *B. arietans*. Proteins with higher unique peptide counts included venom phosphodiesterase in *N. nigricollis* (13 unique peptides), L-amino acid oxidase in *E. ocellatus* (13 peptides), and metalloproteinases in *E. ocellatus* (11 peptides).

The finding that the majority of the identified proteins had low unique peptide count in this study could be due to low peptide yield as a result of the small size of the majority of the identified proteins in this study. Small proteins often generate only 1–2 peptides that fall within the ideal MS detection range [24]. If only one of these peptides ionizes well or meets MS thresholds, a single peptide is identified. Similarly, snake toxins have high protein homologues. This could lead to shared peptide assignment during database search [25].

**Figure 7:**
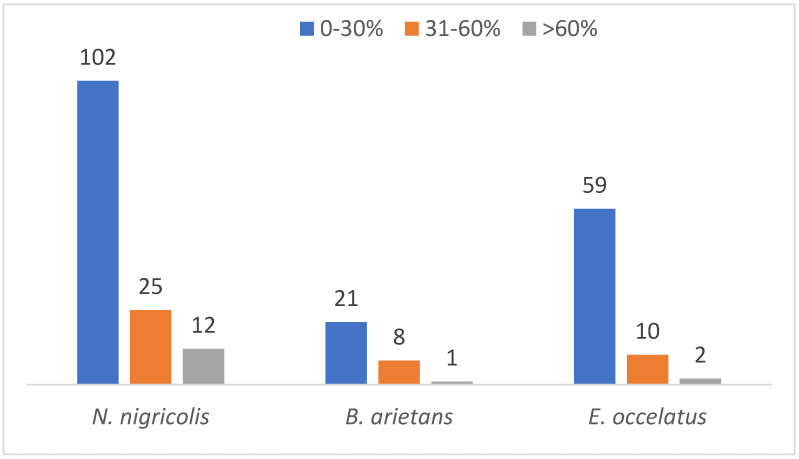
Protein coverage distribution of the identified proteins across the three snake species.

An analysis of protein sequence coverage distribution provided additional insights. The results showed the highest abundance of proteins in the venom of *N. nigricollis*, *B. arietans* and *E. ocellatus* had sequence coverage percentages between 0 and 30% (102, 21 and 59, respectively). This reflects low levels of detection confidence for a significant proportion of the proteome. Only a few proteins were detected to have sequence coverage greater than 60% with 12, 1 and 2 in the venom of *N. nigricollis*, *B. arietans* and *E. ocellatus,* respectively *(*Figure 7).

## 4. Discussion

### 4.1. Proteome Identification

Isobaric tags are isotope-coded molecules with identical chemical structures and molecular weights, consisting of a reporter ion and a balancer. The different isotopes are positioned such that the varying masses of the reporters in a set of reagents are offset by the masses of the balancers. Peptides labeled with isobaric tags display a single peak on an MS spectrum, producing a series of low-mass reporter ions for quantification. The large amounts of MS/MS data generated from quantitative proteomics demand efficient algorithms to process these data, making advanced computational tools essential in the field [26]. iQuant, an automated software tool, facilitates the quantitative analysis of peptides labeled with isobaric tags [27]. It integrates Mascot Percolator and advanced statistical algorithms to process MS/MS signals. At the peptide level, PSMs are pre-filtered at a 1% FDR to ensure high confidence in peptide identification. Using the “parsimony principle,” identified peptide sequences are then assembled into a set of confident proteins. Protein-level FDR is controlled at ≤1% (Protein FDR ≤ 0.01) using the “Picked Protein FDR” strategy during protein inference [27]. In this study, the identified proteins were classified according to toxin families.

Following isobaric peptide tagging and LC-MS/MS analysis, 139, 71, and 30 proteins were identified in the crude venom extracts of *Naja nigricollis*, *Echis ocellatus*, and *Bitis arietans*, respectively (Supplementary Table S1). The identified proteins ranged in molecular mass from 3 to 389 kDa. In total, 240 distinct venom proteins were identified across the three species. These proteins belong to 10, 11, and 88 toxin families in the venoms of *Bitis arietans*, *Echis ocellatus*, and *Naja nigricollis*, respectively.

The venom proteome of *N. nigricollis* was notably complex, with 139 proteins belonging to 88 families. However, most identified proteins in the venom gland of *N. nigricollis* had an average sequence coverage of <10%, indicating low confidence in their discovery. Protein coverage, which reflects the percentage of the protein sequence covered by detected peptides, serves as a key indicator of confidence in protein identification. Higher sequence coverage, supported by an increased number of peptides, enhances the reliability of these identifications [28]. In this study, 3FTx, a core protein family responsible for toxicity in the venom of African spitting cobras (Naja), was found to be the most abundant toxin family. This finding aligns with previous reports indicating a high abundance of 3FTxs and PLA_2_s in the venom of *Naja* species [29]. Interestingly, four “uncharacterized” proteins were detected in the venom gland of *N. nigricollis*. Further analysis is required to determine their sequences and roles; however, their presence suggests the potential for previously unknown functionalities in cobra venom.

Despite the significant differences in the number of proteins identified in each species, all three venoms shared four core proteins: PLA_2_s, SVMPs, SVSPs, and CTLs (Figure 3). These proteins play central roles in the envenomation process, contributing to hemostasis disruption, tissue degradation, and immune modulation [30]. However, the extent of overlap among the venom proteomes of *N. nigricollis*, *E. ocellatus*, and *B. arietans* reflects their evolutionary divergence and functional adaptations. Additionally, *N. nigricollis* venom contained several proteins with no previously known toxic function. Amongst them are globins, actins, annexins, peroxiredoxin, ezrin, triosephosphate isomerase, catenin, and sulfhydryl oxidase. These proteins, while not directly toxic, may play supportive roles in venom function by contributing to stability, protein folding, or interactions with host tissues [14]. Their prevalence in *N. nigricollis* venom suggests that elapid venoms may have evolved to incorporate a broader array of proteins, potentially enhancing venom effectiveness or prey immobilization in unique ways. The identified proteome of *N. nigricollis* indicates that elapid venoms may be more complex than previously understood.

Our finding on the relative distribution of the identified protein families aligns with previous studies that identify metalloproteinases, PLA_2_s, and serine proteinases as the major toxin families in viper venoms [31,32]. However, unlike earlier research by Dingwoke et al. [33], this study revealed a higher abundance of C-type lectins in *B. arietans* venom, with lower proportions of metalloproteinases and serine proteinases. Similarly, metalloproteinases in *E. ocellatus* venom showed higher abundance than previously reported, where SVMPs, SVSPs, C-type lectins, and PLA_2_s were observed at 34.84%, 15.05%, 3.95%, and 21.19%, respectively. These variations may be attributed to differences in transcriptomic activity, as Casewell et al. [34] reported that *B. arietans* venom displays high transcriptional activity of genes encoding C-type lectins, making them predominant during venom secretion.

However, the complexity of the venoms of *Naja nigricollis*, which includes a total of 88 protein families, necessitates a focus on the relative distribution of protein families based on their sequence coverage—a characteristic that underscores the level of confidence in protein detection. A higher peptide count and sequence coverage typically confer greater confidence in protein identifications [28,35]. For this study, proteins with an average sequence coverage greater than 10% were considered. 

In *N. nigricollis*, 22 characterized proteins were identified with >10% coverage, compared to 10 in *E. ocellatus* and 9 in *B. arietans*. C-type lectins, SVSPs, and PLA_2_s were the most abundant protein families in *B. arietans* venom, with relative abundances of 50%, 10%, and 8%, respectively. Metalloproteinases (SVMPs), with an average sequence coverage of only 5%, were not represented (Figure 4A). This does not simply imply the absence of SVMPs in the venom but reflects their low detectability due to insufficient sequence coverage, which may result from their low abundance [36,37].

Similarly, in *E. ocellatus* venom, hyaluronidase, with an average coverage of 9%, was not represented in the pie chart. Nevertheless, metalloproteinases (54%), C-type lectins (17%), and serine proteinases (11%) constituted the most abundant protein families (Figure 4B).

In *N. nigricollis*, 3FTxs were the most abundant toxin family, accounting for 25% of the venom. This was followed by protease inhibitors (cystatins, nawaprins, and serpins) at 8% and PLA_2_s at 7% (Figure 4C). These findings are consistent with previously described proteomic profiles of African spitting cobras, where 3FTxs and PLA_2_s are among the most abundant proteins [20].

These results demonstrate significant differences in the distribution of protein families across the three snake species, reflecting their distinct envenomation strategies and evolutionary adaptations to prey and ecological niches. *N. nigricollis* relies predominantly on neurotoxins like 3FTxs to immobilize prey through paralysis, while *E. ocellatus* and *B. arietans* depend on hemotoxins, with SVMPs and C-type lectins playing pivotal roles in inducing hemorrhage and disrupting blood coagulation [38]. The abundance of PLA_2_s in all three species highlights their multifunctional roles in venom toxicity, contributing to both local tissue damage and systemic effects such as inflammation and anticoagulation [39].

### 4.2. Pathological Mechanisms of the Toxin Families

The 3FTxs found in this study constitute the most abundant protein family in the venom of *N. nigricollis*. These are small, non-enzymatic proteins characterized by their distinct three-finger structural motif. These toxins primarily target receptors involved in neurotransmission, such as nicotinic acetylcholine receptors (nAChRs), leading to paralysis [40]. Alpha-neurotoxins, a major subgroup of 3FTxs identified in this study, bind competitively to nAChRs at the neuromuscular junction, blocking acetylcholine and inhibiting muscle contraction, causing respiratory paralysis and, potentially, death if left untreated [41]. These neurotoxins are divided into short- and long-chain neurotoxins based on the number of amino acids they contain [40]. Long neurotoxins have 60–70 residues, while short neurotoxins have 60 or fewer. Both types prevent the transmission of nerve impulses by competitively inhibiting the binding of acetylcholine to its receptor. Neurotoxins in snake venom are highly effective in quickly immobilizing prey by inducing neuromuscular blockade. Other 3FTxs include cardiotoxins, which disrupt cell membranes, and muscarinic toxins that influence the autonomic nervous system [42].

In contrast, SVMPs—critical components of *E. ocellatus* and *B. arietans*, as identified in this study, are one of the most characterized enzymatic proteins that degrade the extracellular matrix, promoting hemorrhage and local tissue damage by cleaving proteins such as collagen, fibrinogen, and laminin [43]. SVMPs also play a role in the modulation of blood clotting by either activating or inhibiting key factors in the coagulation cascade [44]. In addition to local tissue damage, SVMPs contribute to venom-induced coagulopathy and are involved in prey immobilization through blood loss [45].

PLA_2_s were identified in all three species but with varied relative abundances. These enzymes target phospholipids at the sn-2 position in cell membranes, hydrolyzing them to release lysophospholipids and free fatty acids, including arachidonic acid. This process triggers inflammation and contributes to tissue damage [46]. PLA_2_-induced hydrolysis disrupts the integrity of cell membranes, causing cell lysis and necrosis. The release of arachidonic acid leads to the production of pro-inflammatory eicosanoids, which cause local pain, swelling, and further tissue damage [39]. Some PLA2 isoforms also act as neurotoxins, interfering with neurotransmitter release at neuromuscular junctions. This can result in paralysis, a characteristic effect seen in envenomation by some elapid species [47].

C-type lectins constituted 50% of the proteome in *B. arietans*. These proteins bind carbohydrates in a calcium-dependent manner and interfere with the coagulation cascade by enhancing or inhibiting blood clot formation [48]. Their abundance highlights the anticoagulant properties of *B. arietans* venom. In contrast, *E. ocellatus* and *N. nigricollis* contained lower amounts of C-type lectins, reflecting a divergence in venom functionality [49].

Cysteine-rich secretory proteins (CRiSPs) are non-enzymatic components that interfere with ion channels, including calcium and potassium channels, affecting muscle contraction and neural transmission. CRiSPs can block smooth muscle contraction, promoting prey immobilization by interfering with the normal function of the prey’s musculature [50]. Additionally, CRiSPs are believed to have roles in inhibiting immune responses, thus aiding the venom’s dissemination through the prey’s body.

Another highly studied snake venom toxin family is the SVSPs. These enzymatic proteins play a critical role in the blood coagulation cascade, exhibiting both procoagulant and anticoagulant effects. In their procoagulant role, these enzymes activate prothrombin, fibrinogen, or Factor X, which leads to excessive clot formation and subsequent consumption of clotting factors. This process, known as venom-induced consumptive coagulopathy (VICC), often results in systemic bleeding [51]. On the other hand, certain serine proteases can degrade fibrinogen or inhibit platelet aggregation, preventing clot formation and promoting hemorrhage [52].

The dual effects of serine proteases create a paradoxical condition in envenomation, where localized thrombosis is followed by systemic hemorrhage, exacerbating tissue damage. Additionally, these enzymes modulate components of the kallikrein–kinin system, contributing to vasodilation, increased vascular permeability, and hypotension [53]. This inflammatory response further complicates the toxicological effects of snake venom, leading to more severe systemic issues.

Another protein family found in the venom of *N. nigricollis* is 5′-Nucleotidases. These are enzymes that catalyze the hydrolysis of nucleotide monophosphates into nucleosides and phosphate. These enzymes contribute to the breakdown of cellular ATP, reducing the energy supply available to the prey’s tissues and promoting venom toxicity by inducing cellular dysfunction and death [54]. In addition to their cytotoxic effects, 5′-nucleotidases also enhance the spread of venom through tissue degradation.

L-amino acid oxidases (LAAOs) catalyze the oxidative deamination of L-amino acids, generating hydrogen peroxide as a byproduct. The production of hydrogen peroxide induces oxidative stress, leading to cytotoxic effects on prey tissues [55]. LAAOs also exhibit antimicrobial properties, which may help protect venom components from bacterial degradation. Their role in the venom’s overall toxicity includes inducing cell death, modulating the immune system, and promoting tissue necrosis [56].

Disintegrins are small peptides that inhibit integrin-mediated cell adhesion. They prevent platelets from aggregating, impairing blood clot formation and leading to increased bleeding [57]. Disintegrins are derived from larger snake venom metalloproteinases and contribute to the venom’s ability to disrupt normal hemostasis and promote hemorrhage.

Also found in the venom of *N. nigricollis*, venom nerve growth factors (vNGFs) are proteins that influence the growth and differentiation of nerve cells. In snake venom, NGFs can enhance vascular permeability, facilitating the spread of venom throughout the prey’s tissues [58]. Additionally, NGFs may play a role in modulating the prey’s pain response, though their exact contribution to venom toxicity remains an area of active research.

Glutaminyl cyclase is one of the least understood protein families among various snake venom components. These enzymes act as one of the enzymes for protein post-translational modifications catalyzing the cyclization of glutaminyl residues at the N-terminal of proteins or peptides, forming pyroglutamate residues [59]. Glutaminyl cyclase contributes to the stability or activity of venom peptides [59]. This enzyme family has been associated with enhancing the bioactivity and resistance of venom peptides to proteolytic degradation.

Protease inhibitors such as serpins and venom cystatins are homologous proteins with diverse functions. Serpins are involved in processes such as blood coagulation, fibrinolysis, programmed cell death, development and inflammation [60]. Cystatins, on the other hand, are a group of proteins found in snake venoms that inhibit cysteine proteinases. They bind to and inhibit the activity of cysteine proteases, such as cathepsin L and papain [61].

Lysosomal enzymes, including cathepsins and glycosidases, present in snake venoms, contribute to tissue degradation and prey digestion. Cathepsins (such as cathepsin X and S) are proteases that degrade proteins within lysosomes, promoting tissue necrosis and breaking down structural proteins in prey [62]. Glycosidases, including N-acetylglucosaminidases, break down glycosidic bonds in carbohydrates, further aiding in the digestion of prey tissues. Lysosomal enzymes work synergistically with other proteolytic enzymes, such as metalloproteinases, to enhance tissue degradation and venom spread.

Most of other proteins identified in the venom of *N. nigricollis* have no known toxic function. They can, however, contribute synergistically to the overall toxicity during envenomation. These proteins include globin, actin, annexin, ezrin, peroxiredoxin, glucuronidase, triosephosphate isomerase, catenin alpha, vinculin, cobalamin binding intrinsic factor, ribosomal proteins (large and small subunit proteins) and sulfhydryl oxidase. Protein functional annotation shows that globin proteins are involved in energy production and conservation as part of the heme-containing protein family that includes hemoglobin and myoglobin, which are crucial for oxygen transport and storage in the body [63]. In snake venom, globin is typically not a primary toxin but may be involved in the transport of oxygen within the venom gland or could have a protective role in oxidative stress management. The presence of globin in snake venom is less common and may serve ancillary functions in venom-producing tissues rather than in prey immobilization. Similarly, actin—a highly conserved structural protein involved in the cytoskeleton and cellular movement—was observed in this study. Its presence in snake venom may be indicative of the cellular degradation that occurs when the venom is injected into prey. Actin released from damaged cells in the venom gland or from prey tissues can contribute to the overall tissue-destroying properties of the venom.

Finally, this study provides a detailed comparative analysis of venom proteomes from *Echis ocellatus*, *Naja nigricollis*, and *Bitis arietans* using iTRAQ-based quantitative proteomics. However, several limitations should be acknowledged:

1. Only one iTRAQ experiment was conducted without technical or biological replicates due to sample and cost constraints. Although this may raise concerns about reproducibility, several studies have demonstrated that iTRAQ experiments, when carefully executed, are highly reproducible even in the absence of replicates. Accordingly, Karp et al. [13] and Mertins et al. [14] all reported strong consistency and low technical variability in iTRAQ quantification. Similarly, to enhance confidence in our findings, we implemented rigorous quality control measures, including cross-database searches (SwissProt, PDB, NCBI), stringent peptide/protein FDR thresholds (<1%), and robust data normalization and statistical validation.
2. The bottom-up proteomics approach used in this study effectively identifies a wide range of proteins but may overlook proteoform diversity resulting from post-translational modifications (PTMs) or alternative splicing events. Future studies incorporating top-down proteomics or PTM-specific enrichment strategies may provide a more complete view of venom complexity.
3. Venom samples were pooled by species prior to analysis. While this approach enhances protein representation, it may mask individual or population-level variability in venom composition.
4. Although iTRAQ labeling allows for efficient multiplexing, it may introduce ratio distortion and quantification bias under certain conditions. Nonetheless, our normalization and statistical analysis steps were designed to mitigate these effects.
5. Several proteins, especially from *N. nigricollis* venom, remain functionally unannotated. These proteins require further characterization through functional assays and structural studies to clarify their biological roles and potential therapeutic or toxicological significance.
6. Some protein identifications were based on single unique peptides, which are inherently less robust and more susceptible to changes in database annotation. We addressed this by applying strict FDR control, using high-confidence scores, and focusing on biologically relevant proteins, but these identifications should be validated through targeted proteomic methods such as SRM or PRM.

Future work should aim to incorporate biological replicates, apply complementary top-down proteomics, and perform functional validation to further unravel the complexity and clinical implications of snake venom proteomes.

## 5. Conclusions

The proteomic profiles of three medically significant Nigerian snakes were assessed using label-based MS analysis. The venoms of *E. ocellatus* and *B. arietans* exhibited similar proteomic compositions, showing high relative abundances of C-type lectins, SVSPs, and PLA_2_s. In *E. ocellatus*, these protein families accounted for 17%, 11%, and 5% of the total proteome, respectively, while in *B. arietans*, they constituted 47%, 10%, and 7%, respectively. Although SVMPs were the most dominant proteins in *E. ocellatus*, C-type lectins were the most abundant in *B. arietans*. In contrast, the proteomic profile of *Naja nigricollis* venom revealed that 3FTxs were the most abundant toxin family, with a relative abundance of 9%, followed by metalloproteinases with an intensity value of 54.1 and a relative abundance of 3%. Despite variations in the number of proteins identified in each species, all three venoms shared core proteins from four key families: phospholipases A_2_, metalloproteinases, serine proteases, and C-type lectins. The use of labeling technology in this study was pivotal, uncovering distinct proteins in the venom of *N. nigricollis* that had not been previously identified, including four “uncharacterized” proteins. These findings suggest the potential for novel functionalities in cobra venom. The ability of isobaric labeling to multiplex samples and provide precise quantitative data enabled a comprehensive comparison of venom compositions, offering insights into the functional roles of specific proteins in envenomation. This knowledge is essential for developing targeted antivenoms, highlighting the key proteins responsible for the toxic effects of each species’ venom.

## Figures and Tables

**Table 1:** Identified protein families in the venom of *N. nigricollis*, *B. arietans* and *E. ocellatus.* A more detailed list of the rare protein families is available in Supplementary Table S1.

Abbreviation	Snake Venom Toxin Family	Identification Status			PSC (%)	MM (kDa)
		*E. ocellatus*	*B. arietans*	*N. nigricollis*		
Major toxin families						
PLA_2_	Phospholipase A2	✔	✔	✔	29–46	29–43
SVMP	Snake venom metalloproteinase	✔	✔	✔	5–12	94–2135
SVSP	Snake venom serine protease	✔	✔	✔	16–21	31–210
CTL	C-type lectin	✔	✔	✔	4–29	19–24
3FTX	3-finger toxin	✗	✗	✔	27	78.9
Secondary toxin families						
DI	Disintegrin	✔	✔	✗	30–40	14–21
LAAO	L-amino acid oxidase	✔	✗	✔	21–32	58–113
CRISP	Cysteine-rich secretory protein	✔	✗	✔	18–48	25–29
VEGFs	Vascular endothelial growth factors	✗	✔	✔	2–17	17–48
PIs	Protease Inhibitors	✗	✔	✔	16–27	27–114
Minor toxin families						
NGF	Nerve growth factor	✗	✔	✔	15–30	25–27
5NTD	5′-nucleotidase	✗	✗	✔	34	121
PDE	Phosphodiesterase	✗	✗	✔	9.5	18
PLB	Phospholipase B	✔	✗	✔	24–26	35–64
HYAL	Hyaluronidase	✔	✗	✗	9	52
CYS	Cystatin	✗	✔	✔	8–17	16–29
Rare families (selection)						
QC	Glutaminyl cyclotransferase	✗	✗	✔	34	42
CVF	Cobra venom factor	✗	✗	✔	19	211
Cathepsin		✔	✗	✔	3–10	44–77

Key: ✔ = present; ✗ = absent. Note: proteins were inferred from single peptides and could be vulnerable to database updates and have lower sequence coverage. We applied strict filters (FDR < 1%, and high-confidence scores) and prioritized functionally relevant proteins.

## Data Availability

The original data presented in the study are openly available in iProx at https://www.iprox.cn/page/subproject.html?id=IPX0012188001 (accessed on 5 June 2025), while the Supplementary Table S1 contains the analyzed data from the spectrometry results.

## Supplementary Materials

The following supporting information can be downloaded at: https://www.mdpi.com/article/10.3390/proteomes13030031/s1. Table S1: List of identified proteins and their properties.
